# Alarm Management Competence in Clinical Nursing Practice: An Evolutionary Concept Analysis

**DOI:** 10.1155/jonm/3155022

**Published:** 2026-09-17

**Authors:** Bingyu Li, Liqing Yue, Weizhen Zou

**Affiliations:** ^1^ Teaching and Research Section of Clinical Nursing, Xiangya Hospital of Central South University, Changsha, Hunan, China, csu.edu.cn; ^2^ Xiangya School of Nursing, Central South University, Changsha, Hunan, China, csu.edu.cn

**Keywords:** alarm management, alarm management competence, concept analysis, monitoring systems, nurse

## Abstract

**Aims:**

To clarify the defining attributes, antecedents, and consequences of alarm management competence in clinical nursing practice and to develop a conceptual definition and framework.

**Background:**

Clinical alarms are integral to patient monitoring, yet effective alarm management requires more than technical device operation. Alarm‐related knowledge, skills, judgment, behaviors, and fatigue have been examined across the literature, but the concept of alarm management competence remains insufficiently defined.

**Evaluation:**

Rodgers’ evolutionary concept analysis was conducted. PubMed, CINAHL, Embase, Scopus, and Web of Science were searched from inception to August 2026, supplemented by searches of relevant professional‐organization websites and citation searching. A total of 28 sources were included.

**Key Issues:**

Seven defining attributes were synthesized: foundational clinical knowledge for alarm management, alarm perception and discrimination, clinical decision‐making, technical skills for alarm equipment operation, interprofessional collaboration and communication, alarm fatigue resilience, and professional development capacity. Six antecedents and three domains of potential consequences were identified. These findings were integrated into a conceptual definition and framework of alarm management competence.

**Conclusion:**

Alarm management competence is a dynamic, multidimensional clinical nursing competence that extends beyond alarm‐related knowledge or device operation and integrates cognitive, perceptual, technical, collaborative, resilience‐related, and developmental dimensions.

**Implications for Nursing Management:**

The conceptual framework can inform competency development, education, alarm governance, and organizational support for nurses’ alarm management practice.

**Reporting Method:**

Preferred Reporting Items for Systematic Reviews and Meta‐Analyses Extension for Scoping Reviews (PRISMA‐ScR) checklist.

**Patient or Public Contribution:**

Not applicable.

**Summary**
• What problem did the study address?
◦ Alarm management is a critical component of patient safety, yet the core concept of nurses’ alarm management competence remains poorly defined in the literature.
◦ The lack of conceptual clarity hinders the development of targeted training, assessment tools, and policy interventions.
◦ Current research has not systematically analyzed the attributes, antecedents, and consequences of alarm management competence in clinical nursing practice.
• What were the main findings?
◦ This concept analysis identified seven defining attributes of alarm management competence, as well as six antecedents and three categories of consequences.
◦ Alarm management competence is defined as a dynamic, multidimensional nursing competence shaped by individual, organizational, and environmental factors.
◦ A comprehensive conceptual framework was proposed to guide future research, clinical training, and system‐level improvement.
• Where and on whom will the research have an impact?
◦ The findings provide theoretical foundations for clinical educators, hospital administrators, and nurse leaders to design structured alarm management training.
◦ The findings may support frontline nurses’ skill development and inform efforts to improve alarm‐response practices and address alarm fatigue.
◦ The framework may inform healthcare‐system efforts to strengthen patient safety, optimize care processes, and improve alarm‐related practice.

## 1. Introduction

Clinical alarms (hereafter referred to as alarms) are signals generated by medical devices, such as electrocardiographic monitors, ventilators, infusion pumps, or clinical information systems, to alert healthcare staff to abnormal physiological parameters, equipment malfunctions, or situations requiring clinical intervention [1]. Their primary function is to support the timely detection of potential risks and facilitate prompt clinical action, thereby safeguarding patient safety [2]. With the rapid advancement of healthcare technologies, alarm‐enabled devices have become widely integrated into clinical practice. Consequently, the number of alarms that healthcare professionals must respond to daily has reached a concerning level [3]. False alarms occur more frequently than true alarms [4, 5]. In adult intensive care units (ICUs), each patient may trigger approximately 820 alarms per day, with reported peaks reaching up to 1503 [6]. Repeated exposure to alarms not only weakens the warning function of alarm systems but also contributes to alarm fatigue, increasing the risk of missed alarms, delayed responses, and adverse events [7]. Thus, while alarms are essential to patient safety, they also present substantial management challenges.

Alarm management refers to a set of interventions designed to reduce alarm frequency and improve alarm accuracy, with the goal of preventing alarm fatigue [8]. The quality of alarm management is closely linked to both patient safety and the efficiency of care delivery. When implemented effectively, it can reduce false alarms, facilitate timely responses to critical events, minimize treatment delays, and relieve nurses’ alarm‐related stress [9]. Conversely, inadequate alarm management may result in alarms being ignored, misinterpreted, or inappropriately escalated, thereby increasing clinician workload, delaying necessary interventions, and contributing to adverse patient outcomes [10]. As the primary frontline monitors and responders, nurses play a central role in alarm management. Their clinical judgment and decision‐making shape how nurses interpret alarm signals, determine priorities, and initiate appropriate responses, thereby influencing the safety of patient care [11]. This is particularly apparent in settings characterized by multiple concurrent devices and heavy workloads, where nurses must rapidly interpret alarm priority and clinical significance, coordinate tasks, communicate effectively with team members, and respond under complex conditions [12]. The effectiveness of alarm management, therefore, depends, to a considerable extent, on whether nurses possess the requisite competence.

Competence is commonly understood as an integrated characteristic that enables individuals to combine knowledge, skills, attitudes, and judgment within specific professional contexts in order to achieve intended practice goals [13]. It emphasizes not only the possession of knowledge and skills but also the ability to apply them appropriately in practice and make sound decisions accordingly [14]. In alarm management, nurses require more than familiarity with alarm functions or the mechanical execution of procedures. They must be able to distinguish alarm information, assess clinical risk, determine the timing of intervention, collaborate with team members, and maintain consistent judgment and action under conditions of high pressure and substantial interference [9, 15]. Alarm management should, therefore, be understood not as a discrete technical task but as a complex practice involving cognitive judgment, practical skills, interprofessional collaboration, and contextual adaptation. Accordingly, alarm management competence is best viewed as an integrative nursing construct underpinning effective alarm management practice, rather than as a single technical skill [15, 16]. Given its close association with quality of care and patient safety, alarm management competence warrants systematic attention [10, 17].

In recent years, increasing attention has been paid to nurses’ alarm management competence. Existing studies have approached this topic from multiple perspectives. Some studies have focused on behavioral outcomes, assessing improvements in alarm response performance through quality improvement initiatives [18, 19]. Others have explored cognitive and experiential dimensions, showing how nurses integrate clinical information, experiential judgment, and device understanding during alarm customization [16, 20]. Still others have investigated the contribution of targeted education and training to nurses’ alarm response and management practice [15]. Although these studies offer valuable insights into alarm management practice and potential avenues for improvement, they remain largely problem‐oriented and tend to embed alarm management competence within discrete domains such as knowledge, technical skills, or psychological states. Consequently, limited attention has been given to conceptualizing it as a distinct, integrative, and dynamically evolving form of nursing competence. This has resulted in insufficient clarification of its conceptual boundaries and defining attributes. The lack of conceptual clarity surrounding alarm management competence has limited theoretical advancement and hindered its application in education and training, competence assessment, clinical management, and policy development. Yang et al. have called for the establishment of a standardized assessment framework to evaluate nurses’ alarm management competence comprehensively [10]. However, without a clearly defined concept, the development of assessment instruments and intervention strategies is unlikely to rest on a robust theoretical foundation. Therefore, this study employed Rodgers’ evolutionary concept analysis to examine alarm management competence in clinical nursing contexts, with particular attention to its defining attributes, antecedents, consequences, and surrogate terms and to develop an integrative conceptual definition and framework. By synthesizing dispersed insights from research on alarm fatigue, training, and process improvement, this study aims to provide a theoretical basis for future instrument development and to inform nursing education, curriculum optimization, and the refinement of nurses’ competence evaluation systems.

### 1.1. Aims

This study aimed to apply Rodgers’ evolutionary concept analysis to systematically examine the defining attributes, antecedents, and consequences of alarm management competence. By integrating fragmented insights from the existing literature, the study sought to further clarify its conceptual meaning, theoretical boundaries, and dynamic developmental characteristics and to develop an integrative conceptual framework to inform the development of evidence‐based training programs, the optimization of alarm management practice, and the establishment of nursing competence standards.

## 2. Methods

### 2.1. Study Design

**FIGURE 1:**
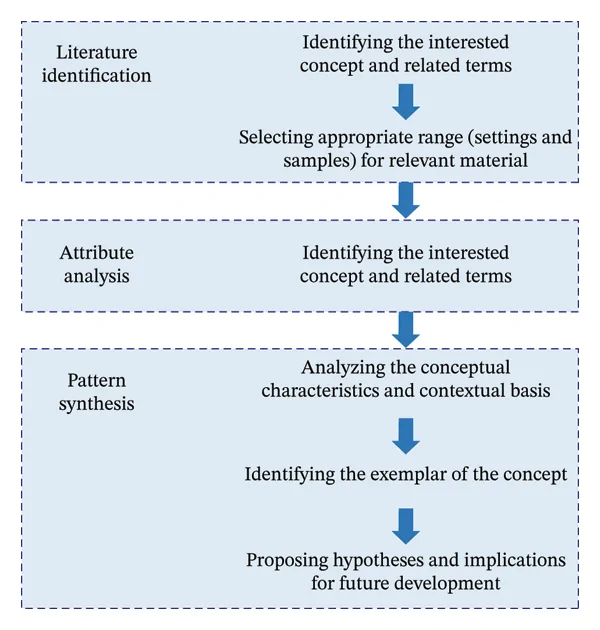
Rodgers’ evolutionary conceptual analysis.

This study employed Rodgers’ evolutionary concept analysis to examine the development of the concept of alarm management competence through systematic literature analysis. In contrast to traditional essentialist approaches, Rodgers’ method emphasizes the contextual and dynamic nature of concepts, making it particularly appropriate for analyzing complex nursing constructs that evolve over time and are shaped by multiple influencing factors in clinical settings [21]. Accordingly, this approach was selected to identify the defining attributes, antecedents, consequences, and contextual manifestations of alarm management competence, with the aim of constructing a multidimensional conceptual framework. The analysis followed Rodgers’ cyclical and iterative process, comprising three phases: literature identification, attribute analysis and pattern synthesis, and across six analytical steps (Figure 1). The review was conducted in accordance with the Preferred Reporting Items for Systematic Reviews and Meta‐Analyses Extension for Scoping Reviews (PRISMA‐ScR) checklist to ensure transparent and rigorous reporting (Supporting Information S1) [22].

### 2.2. Data Sources

To comprehensively examine the use and evolution of the concept of alarm management competence across various contexts, a systematic literature search was conducted. Five academic databases were searched: PubMed, CINAHL, Embase, Scopus, and Web of Science. To supplement the database search, the official websites of relevant professional organizations, including the American Association of Critical‐Care Nurses (AACN), the American Heart Association (AHA), and the International Society for Quality in Health Care (ISQua), were also searched for potentially relevant practice alerts, scientific statements, guidelines, policy documents, and technical reports.

The search strategy combined Medical Subject Headings (MeSH) and free‐text terms using Boolean operators (AND, OR). Core search terms included the following: “clinical alarms” OR “physiologic monitor alarms” OR “alert fatigue, health personnel” OR “alarm fatigue” OR “alarm” OR “alarm management” OR “alarm customization” OR “alarm response” OR “alarm reduce”, “clinical competence” OR “professional competence” OR “competence” OR “competency” OR “nursing skills” OR “knowledge” OR “professional ability” OR “ability.” These terms were selected to capture variations in terminology used in the literature.

All database searches were limited to articles in English. The search covered publications from the inception of each database to August 2026. This approach ensured that the literature captured multiple dimensions of the concept under investigation. The various search strategies used by each database are shown in Supporting Information S2.

Studies were included if they met the following criteria: (1) focused on alarm management competence among healthcare professionals in clinical practice; studies involving non‐nursing professionals (e.g., physicians or technicians) were included only if they provided direct relevance to the conceptual attributes, antecedents, or consequences of nurses’ alarm management competence; (2) involved potential antecedents, surrogates, consequences, definitions, or related terms of the concept; and (3) provided substantive information relevant to the conceptualization of alarm management competence, regardless of study design. Eligible sources included empirical studies, reviews, theoretical or conceptual papers, quality improvement studies, instrument‐development studies, and authoritative professional documents that provided substantive information relevant to the conceptualization of alarm management competence. Studies were excluded if they focused solely on technical aspects such as device engineering or algorithm development without reference to nursing practice.

### 2.3. Data Selection

Following the database search, all retrieved database records were imported into EndNote Version 21 for management and de‐duplication. The screening process was conducted by three members of the research team to ensure consistency and accuracy. In the initial stage, two reviewers independently assessed titles and abstracts against the prespecified inclusion and exclusion criteria. Records were retained when they were judged to be conceptually relevant to the topic of nurses’ alarm management competence. In the second stage, the same two reviewers independently evaluated full‐text articles for eligibility using the same criteria. Sources were included in the final sample if they provided substantive descriptions of the attributes, antecedents, or consequences of alarm management competence. Reference lists of included articles were subsequently hand‐searched to identify additional relevant studies that may have been missed. Documents identified through professional‐organization websites were assessed against the same eligibility criteria. When an eligible document was also available as a formally published article, the published version was retained for inclusion and data extraction. Discrepancies at any stage were resolved through discussion and, when necessary, adjudication by a third senior reviewer, to achieve consensus and ensure a high level of agreement.

### 2.4. Data Extraction and Analysis

Key data were extracted from the included studies by the research team. A standardized data extraction form was developed a priori based on Rodgers’ evolutionary concept analysis framework. Before formal extraction, a small number of articles were randomly selected to pilot test the form. Feedback from the research team was used to refine criteria for identifying attributes and antecedents. When reviews and their underlying primary studies were both included in the analysis, overlap was examined during data extraction. Evidence reported in both sources was not treated as independent or given additional weight because of repeated occurrence. Where overlap was identified, the primary study was used to verify the original context and interpretation of the evidence. Extracted data included the terms used, conceptual definitions or descriptions, defining attributes, antecedents, consequences, related concepts, and contextual descriptions. Information on study design, participant characteristics, and key findings was also recorded. To enhance consistency and accuracy, two reviewers independently coded the data. Following extraction, the coded data were cross‐checked, and any ambiguities were resolved through team discussion or consultation with a third senior reviewer until consensus was reached.

Data analysis was guided by Rodgers’ evolutionary concept analysis [23]. The concept of nurses’ alarm management competence was examined across studies to identify how it was articulated and applied in different research contexts. Through detailed reading and synthesis, the defining attributes were extracted, and both antecedents and consequences were analyzed. Particular attention was paid to contextual variability, including the influence of clinical settings, nurse experience, and types of alarm systems. Thematic synthesis and categorical analysis were employed to integrate data across studies, revealing patterns and changes in the conceptualization of nurses’ alarm management competence. The conceptual model was iteratively refined through team consensus discussions, resulting in an integrated framework encompassing core attributes, related concepts, and implications for practice.

No formal critical appraisal or risk‐of‐bias assessment was undertaken. The primary purpose of this study was to clarify conceptual meaning, identify defining attributes, and map the available evidence, rather than to appraise methodological quality. To capture the dynamic evolution of “alarm management competence” across contexts, diverse sources of evidence were included, spanning theoretical papers and qualitative and quantitative studies, to ensure breadth and depth of the concept analysis.

## 3. Results

### 3.1. Search Results

**FIGURE 2:**
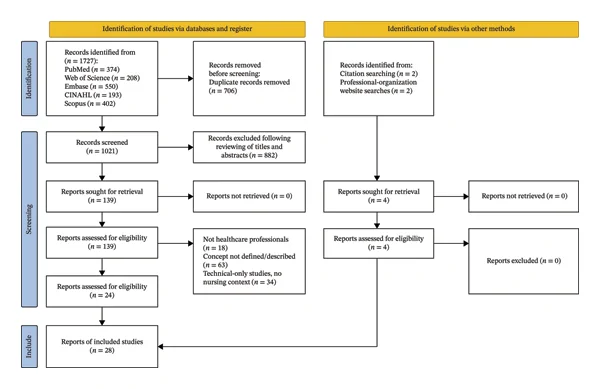
Flow diagram for identification of studies for inclusion in concept analysis.

A total of 1727 records were identified through database searches. After 706 duplicates were removed using EndNote, 1021 records proceeded to title and abstract screening. Of these, 882 were excluded, and the full texts of the remaining 139 articles were assessed for eligibility. Following full‐text assessment, 24 sources identified through database searching met the eligibility criteria. In addition, two records identified through citation searching and two documents identified through supplementary searches of professional‐organization websites were assessed at full text and met the eligibility criteria. In total, 28 sources were included in the concept analysis. The selection process and the number of records at each stage are presented in Figure 2.

### 3.2. Characteristics of the Included Studies

Among the 28 included sources, most were from China (*n* = 11) and the United States (*n* = 11), followed by South Korea (*n* = 2), Türkiye (*n* = 2), Iran (*n* = 1), and Saudi Arabia (*n* = 1). Regarding the source type and study design, nine studies were reviews or evidence syntheses, four were qualitative studies, four were quality improvement or implementation studies, three were cross‐sectional studies, three were quasiexperimental studies, one was a mixed‐methods study, one focused on tool development and educational guidance, one was a single‐group pre–post simulation study, one was a practice alert, and one was a scientific statement.

### 3.3. Attributes of Alarm Management Competence

Attributes are the defining characteristics of a concept [21]. This study identified seven attributes of alarm management competence: (1) foundational clinical knowledge for alarm management, (2) alarm perception and discrimination, (3) clinical decision‐making, (4) technical skills for alarm equipment operation, (5) interprofessional collaboration and communication, (6) alarm fatigue resilience, and (7) professional development capacity. The evidence supporting the identified attributes, antecedents, consequences, and empirical referents across the included studies is presented in Supporting Information S3.

#### 3.3.1. Foundational Clinical Knowledge for Alarm Management

Nurses acquire alarm management knowledge through theoretical education and clinical training, which lays the cognitive foundation for interpreting alarms, making informed decisions, and initiating appropriate interventions [19, 24–26]. This knowledge encompasses multiple domains, including monitoring, disease‐related, and equipment‐related aspects. Specifically, nurses are expected to understand normal monitoring parameter ranges and their clinical implications [12, 20], recognize various alarm types, priority levels, and triggering mechanisms [12, 24, 25], and adjust alarm thresholds according to patient conditions. They should also comprehend the influence of lead selection and parameter configuration on alarm accuracy [18, 20]. Recent evidence further highlights the importance of understanding the underlying causes of alarms and interpreting relevant device parameters and monitoring information in relation to the patient’s clinical condition [27, 28]. Furthermore, proficiency in disease‐specific knowledge related to alarm management is essential, particularly the clinical presentation and monitoring characteristics of conditions associated with high‐risk alarms [24, 29]. For example, nurses should be able to interpret electrocardiograms to identify clinically significant arrhythmias. In addition, they should understand the causes and resolution strategies of common device malfunctions and continuously improve their system‐level knowledge through participation in manufacturer‐led training and ongoing professional education [18, 24].

#### 3.3.2. Alarm Perception and Discrimination

Alarm perception and discrimination are prerequisites for initiating clinical alarm management, involving nurses’ sensitivity to alarm signals and their ability to make initial judgments about alarm classification [12, 16, 25, 29–31]. Nurses must accurately identify the source, type (physiological/technical), and priority of alarms based on sound characteristics, distinguish clinically meaningful signals from nonactionable alarms, and interpret their significance in relation to the patient’s condition when determining response priority [12, 18, 32–34]. Perceptual ability is influenced by the frequency of false alarms, with frequent false alarms reducing nurses’ ability to recognize clinically meaningful signals [12, 31, 32]. This ability can be enhanced through simulation training and continued exposure to alarm environments, forming the foundation for subsequent clinical decision‐making [15].

#### 3.3.3. Clinical Decision‐Making

Clinical decision‐making involves nurses assessing alarm signals in the context of patient status, treatment goals, and alarm content to prioritize actions, select intervention pathways, and adjust parameters accordingly [20, 25]. Research indicates that nurses must distinguish high‐risk signals from multiple alarms and make appropriate decisions, including clinical interventions, technical troubleshooting, or collaborative responses [8, 25, 32]. Such decisions require nurses to interpret the clinical significance of alarms in context, prioritize risks, and determine whether bedside intervention, continued assessment, technical troubleshooting, or escalation is warranted [34, 35]. This competence relies on nurses’ understanding of disease progression, ability to assess individual differences, and capacity to integrate information under pressure [12, 18, 20, 29]. Clinical decision‐making also involves timely adjustments to parameters and clear role delineation, reflecting nurses’ proactive judgment and action in complex healthcare systems [20, 24, 25, 29]. For novice nurses, decision‐making efficiency depends on standardized decision‐making processes and early clinical practice experience [36].

#### 3.3.4. Technical Skills for Alarm Equipment Operation

Proficiency in equipment operation is the foundation for translating nurses’ alarm‐related knowledge into effective clinical actions. These technical skills encompass routine handling of monitoring devices, individualized alarm threshold setting and adjustment, maintenance of appropriate sensor or device connections, troubleshooting common malfunctions, coordinating multiple systems, and responding flexibly in time‐sensitive or high‐acuity situations [19, 24, 25, 37, 38]. Nurses are expected to perform essential operations such as initiating or silencing alarms, adjusting parameters, and updating patient information to ensure uninterrupted physiological monitoring [8, 24, 25]. When faced with technical alarms, they should be able to independently carry out basic troubleshooting—such as checking electrode connections or replacing leads—after identifying possible device malfunctions [18, 20, 24, 25, 33]. In settings with multidevice systems, nurses need to integrate information from both bedside and central monitors, ensuring alarm sources are clearly identified and data remain synchronized [18, 24, 25]. During patient transport or emergencies, they should demonstrate the ability to swiftly switch alarm modes, confirm power and connectivity status, and locate core monitoring equipment, thereby ensuring the continuity of essential parameter tracking [19, 37].

#### 3.3.5. Interprofessional Collaboration and Communication

Interprofessional collaboration and communication are essential components of alarm management, reflecting nurses’ involvement in goal setting, role division, information feedback, and process optimization within multidisciplinary teams [19, 32, 37]. Nurses should collaborate with team members to set individualized alarm thresholds, offer suggestions based on patient conditions and treatment goals, and promote precise alarm parameter management [19, 20]. Nurses must clarify their responsibilities, lead daily alarm monitoring and initial responses, promptly report changes in patient status to physicians, and collaborate with engineers to address equipment abnormalities, ensuring system efficiency [20, 30, 32, 37]. This also requires nurses to recognize professional role boundaries and escalate alarm‐related problems when further decisions or interventions fall outside their scope of practice [34]. In terms of information sharing, nurses should use standardized tools to report frequent alarm occurrences and share handling experiences, facilitating interprofessional understanding and reassessment of alarm data [18, 36]. Additionally, nurses should actively engage in interprofessional communication and experience transfer to promote team sharing and continuous optimization of critical alarm identification and response practices [12, 29, 32, 39]. Nurses also act as a communication bridge with patients and families, explaining alarm mechanisms, alleviating anxiety, and fostering a supportive alarm response environment [12].

#### 3.3.6. Alarm Fatigue Resilience

Alarm fatigue resilience refers to a nurse’s ability to maintain responsiveness and professional composure under sustained exposure to frequent alarms [12, 19]. It involves emotional regulation, cognitive load control, and consistent response to critical alarms, even in the presence of repetitive, low‐priority, or invalid signals [12, 25]. This resilience may help nurses maintain effective alarm responses despite prolonged exposure to frequent alarms and is associated with accurate alarm interpretation and adaptation to complex clinical environments [15, 29]. Nurses with greater resilience and self‐regulation are better equipped to stay focused and manage stress in high‐pressure environments [12]. Team‐based supports, such as fair scheduling, shared experiences, and alarm system optimization, also contribute to individual resilience and stress adaptation [32, 37]. When nurses can effectively regulate their state, they are more likely to sustain alarm management performance, reinforcing their alarm management competence over time [12, 19]. Alarm fatigue resilience should, therefore, be distinguished from reduced alarm fatigue: the former concerns the maintenance of alarm‐related attention, judgment, and response under sustained alarm exposure, whereas the latter describes the level of fatigue experienced [38, 40].

#### 3.3.7. Professional Development Capacity

Professional development capacity refers to nurses’ ongoing ability to learn and adapt within evolving alarm management systems. It encompasses knowledge renewal, skill enhancement, and responsiveness to institutional changes [12, 19, 36, 37]. As technologies advance through smart devices, improved alarm algorithms, and redesigned clinical workflows, nurses need to update and apply alarm‐related knowledge and skills and adapt their practice to changing clinical and technological requirements [12, 24, 36, 37]. This capacity involves not only the adoption of new technologies and practices but also their continued integration into alarm management as clinical and organizational contexts change [19, 40]. Professional development capacity is, therefore, distinct from participation in education or training, which may support competence development, and from professional development as a potential outcome of enhanced competence.

### 3.4. Antecedents of Alarm Management Competence

Antecedents are defined as events or conditions that contribute to the emergence of a concept [21]. This study identified six key antecedents that influence the development of alarm management competence: (1) nurses’ individual traits and motivation, (2) education and training, (3) clinical practice and experience accumulation, (4) organizational and team environment, (5) equipment and technological infrastructure, and (6) human resources and work environment.

Nurses’ individual traits and motivation serve as intrinsic drivers in the development of alarm management competence [8, 20, 33, 41]. A strong motivation for learning and professional improvement, together with a proactive learning attitude, promotes engagement in training and the accumulation of clinical experience, thereby enhancing alarm management competence [19, 33]. Furthermore, when nurses recognize the risks that alarm fatigue poses to patient safety and their own well‐being, they are more inclined to improve their response strategies and increase management effectiveness [8, 19, 20, 29].

Education and training, driven by nurses’ intrinsic motivation, are key to developing alarm management competence [12, 15, 16, 42]. Structured onboarding programs help establish foundational knowledge of device operation [19, 24]. Sowan et al. reported that vendor‐led training on physiological monitors during orientation supported nurses’ later understanding of alarm systems. Continuing tailored education, individualized instruction, and simulation‐based training may enhance nurses’ ability to manage complex alarms in clinical settings [12, 15, 18, 25]. More recent studies have further supported structured, simulation‐based, and peer‐led approaches to alarm management education, with improvements reported in alarm‐related knowledge, behavioral intentions, clinical competence, and alarm management behaviors [27, 38, 40]. Lu et al. highlighted that such approaches significantly improve nurses’ alarm management competence in personalized alarm settings and prioritization [12]. Peer‐led education and guidance from experienced nurses can support knowledge transfer and the development of alarm‐management knowledge and behavioral intentions [25, 36, 40].

Accumulated clinical experience plays a fundamental role in developing alarm management competence [20, 29, 33]. With increasing years of practice, nurses become more skilled in distinguishing alarm types and selecting appropriate response strategies [8, 25, 33, 34]. This advantage is particularly evident in high‐alarm settings such as ICUs [12, 37, 39]. Additionally, specialty‐specific backgrounds contribute to differences in alarm‐handling skills [20]. For instance, neuro ICU nurses are more experienced in managing intracranial pressure alarms, while surgical ICU nurses are more proficient in responding to transport monitor alarms—both shaped by repeated exposure within their specific clinical contexts [25].

Standardized alarm management policies and procedures at the organizational level provide a structural foundation for nursing practice [18, 19, 24, 32, 33]. Interprofessional collaboration enhances nurses’ understanding of monitoring goals and intervention strategies, thereby strengthening communication and coordination [18, 19, 24]. Additionally, experienced nurses who serve as “super users” offer operational guidance, promoting both collective skill development and a culture of team‐based knowledge sharing [12, 20, 25, 33].

Supportive alarm system designs, including intuitive interfaces, clear waveform displays, and distinct priority indicators, provide an important environmental foundation for effective alarm recognition and response [25, 29, 33]. High‐reliability devices help reduce false alarms and cognitive burden, thereby improving overall efficiency [8, 19, 33]. Technical support from engineers and the integration of intelligent alarm systems further equip nurses with tools to optimize response processes [8, 18, 29, 34].

Adequate human resource allocation and a supportive work environment serve as essential foundations for the development of nurses’ alarm management competence [15, 37]. Sufficient staffing, reflected in balanced nurse‐to‐patient ratios and well‐structured shift schedules, provides the necessary time and capacity for focused and accurate alarm management, reducing the likelihood of task shortcuts under workload pressure [20, 33, 39]. Environmental improvements, such as minimizing noise and optimizing equipment layout, further reduce barriers to alarm recognition and response, facilitating more efficient alarm handling [20, 29, 32, 39].

### 3.5. Consequences of Alarm Management Competence

Consequences refer to outcome events that may arise following the development of a concept [21]. In the literature reviewed, alarm management competence was conceptually associated with outcomes concerning nurses, patients, and healthcare systems. These potential consequences can be categorized into three major domains: nurse benefits (reduced alarm fatigue, improved physical and mental well‐being, and enhanced professional development), patient safety (increased protection, improved recovery, and enhanced trust), and system optimization (improved workflows, increased operational efficiency, and enhanced institutional reputation).

Nurses with strong alarm management competence may be better positioned to manage alarm fatigue and maintain psychological and physical well‐being in clinical practice [18, 19, 33]. By accurately identifying alarms and applying appropriate management strategies, such as eliminating redundant alerts and configuring suitable pause settings, nurses may experience a lower cognitive and emotional burden associated with ineffective alarms [25, 29, 39]. In high‐alarm clinical environments, nurses with lower levels of alarm management competence may be more vulnerable to fatigue and reduced responsiveness, whereas those with stronger alarm management competence are more likely to sustain alertness and functional resilience [24]. The literature also suggests potential associations with job satisfaction and a stronger sense of self‐efficacy, which in turn enhances motivation for continuous learning and professional engagement [19, 25]. Over time, involvement in alarm management practices may be associated with the development of alarm‐related knowledge, clinical judgment, and technical proficiency, as well as psychological resilience and self‐regulation in high‐pressure situations [12, 19].

Alarm management competence among nurses has been conceptually linked to patient safety and related care outcomes [15, 30]. Accurate and prompt responses to critical alarms may help reduce the risk of adverse events associated with misinterpretation or delayed intervention [8, 15, 20, 32]. For instance, early detection and resolution of technical alarms, such as electrode dislodgement, help maintain uninterrupted monitoring and ensure the reliable acquisition of vital signs for timely clinical actions [30, 36]. In addition, effective alarm management reduces unnecessary environmental noise and distractions, potentially contributing to a quieter and safer therapeutic setting that supports patient rest and recovery [12, 18]. Reliable alarm responses may also support earlier identification of clinical deterioration and timely clinical intervention, with potential implications for patient recovery [24, 36, 39]. Furthermore, high alarm management competence may be associated with greater patient and family trust and satisfaction, contributing to a more positive care experience.

From a systems perspective, stronger alarm management competence may be associated with more efficient clinical workflows [25, 32, 37]. By minimizing the disruption caused by false or nonactionable alarms, healthcare professionals are better able to concentrate on high‐risk events and priority patients, potentially supporting more focused and efficient resource allocation [39]. Accurate alarm data may also support interdisciplinary collaboration and information sharing, contributing to the development of cohesive and responsive care teams [8, 12]. In addition, robust alarm management supports greater continuity and responsiveness in care delivery and advances the standardization of patient safety practices. Collectively, these proposed consequences may extend to service quality and operational performance; however, evidence for broader outcomes such as institutional reputation and public trust remains limited.

**FIGURE 3:**
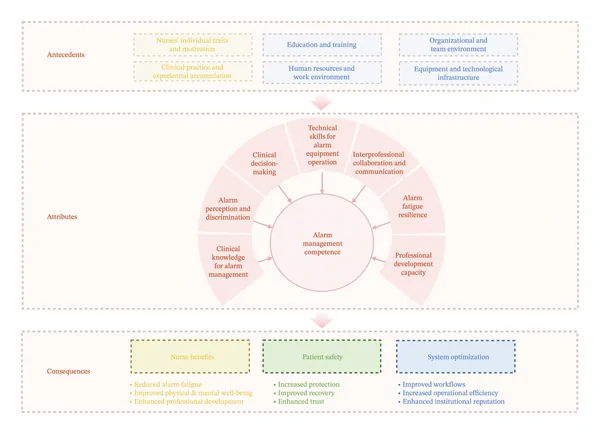
Conceptual model of nurses’ alarm management competence. *Note:* The links among the elements in the figure represent conceptual and associative relationships synthesized from the literature, rather than statistically inferred causal relationships.

Building on the above concept analysis, a conceptual model of nurses’ alarm management competence was developed (Figure 3) to depict the relationships among antecedents, defining attributes, and consequences. The model indicates that antecedent conditions, including education and training, clinical experience, and organizational support, may contribute to the formation and development of alarm management competence and support the coordinated development of the seven core attributes. These attributes are integrated within nurses’ alarm management competence and are conceptually associated with potential outcomes across nurse benefits, patient safety, and system optimization. These relationships represent conceptual associations identified from the literature rather than established causal effects.

### 3.6. Definition of Alarm Management Competence

The reviewed literature presents varying descriptions of alarm management competence, which are summarized in Supporting Information S4. However, no existing definition comprehensively encompasses all the attributes identified in this analysis. Accordingly, based on the synthesized attributes, the following definition is proposed.

Alarm management competence is a dynamic and multidimensional clinical nursing competence, grounded in systematic alarm‐related knowledge and technical proficiency, and manifested through accurate alarm perception and discrimination, contextually appropriate clinical decision‐making, effective interprofessional collaboration and communication, resilience to sustained alarm exposure, and continuous learning and adaptation to evolving clinical and technological contexts.

To ensure conceptual consistency, the alignment between the seven identified attributes and their corresponding operational elements within the definition is summarized in Table 1.

**TABLE 1:** Mapping of identified attributes to operational elements and conceptual roles.

Identified attributes	Operational elements in definition	Conceptual role
Foundational clinical knowledge for alarm management	Systematic knowledge	Cognitive base
Alarm perception and discrimination	Alarm perception	Sensory trigger
Clinical decision‐making	Critical judgment	Reasoning core
Technical skills for alarm equipment operation	Technical proficiency	Psychomotor execution
Interprofessional collaboration and communication	Interprofessional collaboration	External support
Alarm fatigue resilience	Psychological resilience	Internal regulation
Professional development capacity	Proactive professional development	Continuous evolution

### 3.7. Surrogate Terms and Related Terms

Surrogate terms refer to alternative terms that have been used to describe a concept and may overlap in meaning in specific contexts [21]. In this study, the core term is alarm management competence. The review of the literature identified several surrogate terms, including “alarm management ability” [12, 16, 33], “alarm management skills” [12, 19, 20, 24, 25, 29, 37], “alarm management competency” [18, 19, 37], and “alarm management capability” [12]. In the Physiologic Monitor Use and Alarm Management Guide developed by Phillips et al. alarm management competence is conceptualized as an integrated construct encompassing knowledge, skills, clinical reasoning, and attitudes. Within this framework, competence is further described through three core components: knowledge, skills, and clinical reasoning. Although these terms have occasionally been used interchangeably in previous studies, they represent related but conceptually distinct expressions that reflect overlapping aspects of applied proficiency and clinical judgment within alarm management practice.

Among these, “alarm management skills” tends to emphasize procedural execution and task‐level proficiency. However, this term does not fully capture the multidimensional nature of alarm management competence. A nurse may be technically proficient in performing isolated alarm‐related tasks with limited theoretical knowledge and reasoning capacity. In contrast, alarm management competence requires the integration of knowledge, clinical judgment, situational assessment, interprofessional coordination, and active participation in system‐level improvement efforts. Therefore, competence is conceptually broader than skills.

Related concepts, while not synonymous, are closely associated with the target term. These include “alarm management” [8], “alarm management practice” [15], and “alarm management behavior” [29, 39]. Each of these terms addresses distinct but complementary dimensions of the concept, including interventions, implementation strategies, and observable behaviors. Together, they form an interconnected framework surrounding alarm management competence.

Overall, although these surrogate terms each emphasize particular aspects of alarm management, “alarm management competence” represents a more integrative core concept that brings these perspectives together within a single conceptual framework. It extends beyond the procedural execution implied by “skills” and differs from the related notion of “capability,” which generally emphasizes potential performance within a given context. Instead, it encompasses higher‐order elements such as prudent judgment, interprofessional collaboration, and psychological resilience. By integrating technical proficiency, clinical judgment, and the capacity to adapt to complex contexts, the proposed definition offers a more comprehensive perspective that attends not only to observable behavioral performance but also to the cognitive foundations, collaborative processes, and organizational support required to achieve effective alarm management in complex clinical environments.

### 3.8. Model Case

Hu, a 28‐year‐old nurse with five years of experience in a cardiovascular unit, regards alarm management as essential to patient safety and consistently integrates standardized response procedures into her daily practice (individual traits and motivation). Her unit conducts quarterly training, provides smart monitoring equipment, and uses flexible scheduling (education and training, organizational and team environment, equipment and technological infrastructure, and human resources and work environment). Recently, she began studying tiered alarm response protocols to improve efficiency (clinical practice and experience accumulation).

One day, a middle‐aged male patient returned to the ward after undergoing emergency percutaneous coronary intervention (PCI) for an acute inferior myocardial infarction. Prior to this, Nurse Hu had already provided education on alarm‐related knowledge to the patient and his family. Two hours postprocedure, a ventricular fibrillation alarm sounded. Though other low‐priority alarms were active, she quickly recognized the high‐priority signal (foundational clinical knowledge for alarm management and alarm perception and discrimination), assessed the patient’s condition, and judged it as a true emergency (clinical decision‐making). She initiated defibrillation (technical skills for alarm equipment operation) and coordinated with the medical team (interprofessional collaboration and communication). Despite initial defibrillation failure and the ongoing alarm noise, she remained focused (alarm fatigue resilience) and successfully restored sinus rhythm on the second attempt. Later, she proposed a family‐engaged alarm response guideline for post‐MI care (professional development capacity). This case illustrates the impact of alarm management competence: it enhanced nurse performance (nurse benefits), ensured timely patient intervention (patient safety), and supported system‐level improvements in alarm response processes (system optimization).

### 3.9. Empirical Referents and Potential Operationalization of Alarm Management Competence

Empirical referents describe how a concept may be recognized or observed in practice. In this study, they are presented as potential operational manifestations rather than validated measurement criteria. Potential approaches for examining alarm management competence include four broad domains: assessments of knowledge and rule‐based judgment, objective performance information derived from scenario‐based simulation or usability testing, structured observation and process evaluation, and questionnaire‐based assessments.

For foundational clinical knowledge, alarm perception and discrimination, and clinical decision‐making, empirical referents are best reflected in knowledge tests and scenario‐based tasks. These assessments can focus on measurable performance such as alarm identification and prioritization, the appropriateness of alarm threshold setting and adjustment, selection of response pathways, and escalation timing [43]. Educational and evaluation resources grounded in a knowledge, skills, and attitudes framework can inform assessment content and performance standards [24, 44]. In addition, simulation and usability‐testing studies provide a useful framework for translating recognition performance, decision quality, and operational execution into objective metrics. Performance parameters such as response time, error patterns, and task completion may provide objective information regarding specific components of alarm management performance [20, 45]. For technical skills related to alarm device operation, existing structured instruments offer more direct empirical referents. For example, the Nurse Competence on Physiologic Monitors Use Survey covers domains including hardware and connectivity management, alarm management, appropriate monitoring, and advanced function use [25].

For interprofessional collaboration and communication, empirical referents can be drawn from structured observation indicators of standardized communication behaviors and coordinated response processes. Survey instruments assessing alarm‐related attitudes and practices can further describe alarm management at the team level [19, 46, 47]. For alarm fatigue resilience, alarm fatigue questionnaires and brief versions of such tools can serve as key referents and can be combined with performance indicators such as the maintenance of response rates to critical alarms and delayed response rates under high alarm load to reflect the capacity to maintain response quality during prolonged exposure. For professional development capacity, process and outcome evidence is likely to be more appropriate, including participation records for continuing education and training, competency achievement following implementation of new devices or workflows, the frequency of engagement in process improvement and knowledge sharing, and performance improvement trajectories over time [43].

Overall, existing evidence provides direct or indirect empirical referents for some attributes of alarm management competence. However, systematic measurement of this integrative construct remains limited, and coverage across attributes is uneven.

Therefore, illustrative manifestations were further synthesized for each attribute (Table 2) to support education and training, future competence assessment, and subsequent instrument development.

**TABLE 2:** Illustrative manifestations for operationalizing the core attributes of nurses’ alarm management competence.

Attributes	Illustrative manifestations
Foundational clinical knowledge for alarm management	Knowledge demonstrated through structured assessments of alarm types, priority levels, threshold principles, common technical causes, and initial management strategies; clinical reasoning related to alarm parameter adjustment in case‐based scenarios
Alarm perception and discrimination	Ability to identify and differentiate clinically meaningful alarms from nonactionable signals in simulated or observed alarm scenarios, including recognition of alarm sources, priorities, and potential clinical significance
Clinical decision‐making	Ability to prioritize alarms, integrate patient conditions with alarm information, and select appropriate response pathways and escalation strategies in complex clinical scenarios
Technical skills for alarm equipment operation	Demonstration of appropriate operation of monitoring equipment, including alarm configuration, parameter adjustment, alarm acknowledgment, restoration, and basic troubleshooting procedures
Interprofessional collaboration and communication	Effective communication and coordination during alarm management, including structured information exchange, escalation processes, and collaboration with multidisciplinary team members
Alarm fatigue resilience	Maintenance of appropriate alarm responsiveness and clinical judgment under conditions of high alarm exposure, together with adaptive strategies for managing cognitive and emotional burden
Professional development capacity	Engagement in continuing education, reflective learning, and improvement activities related to alarm management, including adaptation to evolving technologies and clinical practices

*Note:* These manifestations illustrate how alarm management competence may be expressed in practice; they are not validated measures or scoring criteria.

## 4. Discussion

This study applied Rodgers’ evolutionary concept analysis to clarify the defining attributes, antecedents, and consequences of alarm management competence in clinical nursing practice and developed a conceptual definition and framework to support theoretical development and practical application in this field [23]. The study’s conceptual contribution lies in bringing together alarm‐management components that have been addressed across different strands of previous research and integrating them into a coherent clinical nursing construct with clearer meaning and boundaries. Seven defining attributes were synthesized within this conceptual framework: foundational clinical knowledge for alarm management, alarm perception and discrimination, clinical decision‐making, technical skills for alarm equipment operation, interprofessional collaboration and communication, alarm fatigue resilience, and professional development capacity. While individual studies may emphasize different aspects, these attributes collectively reflect the multidimensional and evolving nature of alarm management competence in complex and dynamic alarm environments.

During the literature analysis, six key antecedents were identified as critical to the development of alarm management competence: nurses’ individual traits and motivation, education and training, clinical practice and experience accumulation, organizational and team environment, equipment and technological infrastructure, and human resources and work environment. These antecedents are interconnected and may collectively contribute to the development of alarm management competence. From an internal logic perspective, individual characteristics and motivation serve as core driving forces for the development of alarm management competence [48]. Nurses’ willingness to learn facilitates engagement in structured training, which provides a foundation for clinical practice [40]. Training builds a knowledge base that is subsequently refined through hands‐on experience [40]. As clinical exposure increases, theoretical understanding evolves into context‐specific alarm management competence [49]. Unlike novice nurses, who tend to rely more heavily on standardized procedures, experienced nurses are better able to rapidly extract salient information and exercise judgment in complex alarm situations [12]. Organizational support, such as training opportunities, collaborative teams, and advanced technology, further safeguards the conversion of experience into competence development [29, 38].

The Patient Risk Detection Theoretical Framework (PRDTF) offers a conceptual lens for interpreting these dynamics [50]. The framework proposes that, in complex environments, the effectiveness of nurses’ alarm responses depends on their ability to discriminate true risk signals from irrelevant stimuli and to prioritize accordingly [50]. This competence is shaped through the combined influence of the aforementioned antecedents. Among these factors, intrinsic motivation appears particularly important. Compulsory training or passive participation may fall short of fostering meaningful learning [51]. Alarm management competence should, therefore, be nurtured through strategies that engage nurses’ internal drive, enabling them to select educational opportunities aligned with their professional goals and perceived learning needs. Based on these insights, future research should focus on how personalized education and peer‐based experience sharing can bolster novice nurses’ confidence and decision‐making capacity.

The proposed consequences of alarm management competence span three main areas: nurses’ wellbeing and professional development, patient safety and care experience, and healthcare system performance. At the nurse level, stronger alarm management competence has been associated with lower alarm fatigue, sustained work performance, and greater professional confidence [19, 33]. At the patient level, competence in recognizing, interpreting, and responding to alarms may support timely clinical decisions and has been conceptually linked to patient safety and the care environment [20, 29]. While previous research has focused primarily on immediate nurse responses and patient safety‐related outcomes, broader system‐level associations have received less attention [10, 36]. Potential system‐level consequences identified in the literature include more focused resource allocation, interdisciplinary collaboration, and continuity of care [12, 14]. These findings suggest possible associations across nurse‐, patient‐, and system‐level outcomes, but the available evidence does not establish a causal pathway between alarm management competence and these consequences.

At the same time, the formation and development of alarm management competence continue to face practical barriers, including attentional fragmentation associated with heavy workloads and operational complexity arising from sophisticated device design [52, 53]. Research has shown that nurses’ ability to detect clinical warning signals is not solely determined by their alarm management competence but is also shaped by contextual factors within the work environment [54]. The PRDTF highlights that organizational structures, dynamic workflows, and safety culture can either support or undermine nurses’ ability to perceive and act upon alarm cues [50]. For example, excessive background noise in ICU settings may impair nurses’ discrimination of alarm signals, while staffing shortages and sustained high workload may reduce vigilance and resilience to fatigue [55, 56]. Accordingly, healthcare organizations should draw on analyses of alarm‐related adverse events to identify modifiable factors across environmental optimization, staffing and scheduling, and system‐level supports, thereby strengthening the organizational foundations that enable the development of alarm management competence.

Furthermore, this competence demonstrates clear contextual adaptability and applicability across clinical settings. Although the literature has examined alarm management extensively, relatively limited attention has been paid to its context‐sensitive and developmental characteristics. Clinical practice suggests that nurses from different specialties exhibit distinct patterns of alarm management, indicating that competence development is shaped jointly by professional background and practice setting [57, 58]. While alarm management is often discussed in relation to ICUs where alarm burden is high, the core attributes identified in this study are not confined to ICUs. They are also relevant to non‐ICU settings equipped with monitoring systems, including general wards, perioperative units, and emergency observation units. In such environments, where alarms may occur less frequently but may carry greater clinical warning value, timely perception and accurate judgment of critical alarms are essential for reducing the risk of delayed recognition and management of patient deterioration [50]. As clinical contexts vary, the ways in which the attribute of interprofessional collaboration and communication is expressed may also differ. In ICUs, alarm management is typically embedded within continuous monitoring and rapid escalation processes, placing greater emphasis on intensive team coordination around critical‐alarm recognition and immediate intervention. In non‐ICU settings, alarm management may rely more on coordinated work between nurses, physicians, engineering and technical staff, and other relevant professionals to support detection of deterioration, escalation and follow‐up, and device‐related assistance.

In addition, when considering the transferability of alarm management competence, cross‐cultural and system‐level differences warrant careful attention. Variations across countries and regions in staffing models, access to technology, alarm management policies and procedures, professional role boundaries, and patient safety culture may all shape how this competence is enacted in practice. In healthcare systems characterized by stronger technology integration and higher levels of process standardization, alarm management competence may rely more heavily on formal training, technological integration, and team‐based coordination. In contrast, in relatively resource‐constrained environments, nurses may depend more on accumulated clinical experience, intuitive judgment, and informal peer support to manage alarm‐related risks [25, 39]. Accordingly, although the core attributes identified in this study have broad relevance, their specific manifestations and practical application should be interpreted in relation to local healthcare contexts and adapted as appropriate.

Existing instruments also capture several dimensions identified in this analysis. The Nurse Competence on Physiologic Monitors Use Survey assesses nurses’ competence in the use of physiologic monitors, including alarm‐related functions, while other instruments have assessed alarm‐related knowledge, skills, attitudes, and management practices [24, 25]. These measures provide useful assessments of specific aspects of alarm management; however, they do not necessarily encompass alarm management competence as the broader multidimensional construct conceptualized in this study. In particular, the present analysis brings together cognitive, technical, perceptual, collaborative, resilience‐related, and developmental dimensions within a single conceptual framework. Recent alarm‐management studies similarly continue to focus on particular aspects of practice, including education, alarm‐setting practices, response behaviors, and alarm fatigue. These studies provide further support for several components represented in the present framework, particularly alarm‐related knowledge and skills, clinical decision‐making, and education as an antecedent. At the same time, their focus on specific interventions or outcomes highlights the value of considering these elements together as part of a broader competence construct.

Alarm management competence should not be viewed solely as an individual nurse‐level construct. Alarm parameter optimization, clinical interpretation, escalation, and troubleshooting involve interprofessional practice shaped by team communication, role delineation, and shared workflows. The proposed framework, therefore, provides a structured lens for understanding alarm management competence as an integrative nursing competence rather than a set of technical tasks or observable behaviors.

### 4.1. Limitations

This concept analysis has several limitations. First, the cross‐cultural applicability of the findings requires further evaluation. Most included studies were conducted in the United States and China, whereas only a small number were conducted in Iran and Turkey. Differences across healthcare systems in staffing models, technological resources, alarm management policies, professional role boundaries, and safety culture may influence how the concept is understood and applied across settings. Future research should examine the framework in a wider range of countries and regions. Second, the analysis focused exclusively on nurses, without addressing alarm management competence among other healthcare professionals such as physicians or respiratory therapists, although alarm management is inherently interprofessional. Third, many included studies were qualitative or descriptive in nature, limiting the ability to examine relationships among attributes and outcomes. Future empirical investigations, including the development of standardized measurement instruments and structural equation modeling, are needed to further examine these relationships.

### 4.2. Implications for Nursing Management

Alarm management competence should be incorporated into nursing quality and safety management rather than treated solely as an individual nurse responsibility. Nursing managers should establish clear policies for alarm settings, escalation, response, and review, while ensuring appropriate staffing and workload arrangements to support timely responses to clinically important alarms. Regular review of alarm patterns, nonactionable alarms, and alarm‐related adverse events can help identify problems in alarm parameters, equipment, and clinical workflows. Unit‐based alarm resource nurses or “super‐users” may provide bedside guidance, peer support, and feedback on alarm‐management practices.

Competency development should extend beyond initial device training and include clinical reasoning, alarm prioritization, individualized parameter setting, and strategies for managing alarm fatigue. Simulation, refresher training, competency reassessment, and mentorship can be incorporated into orientation and continuing professional development according to nurses’ roles and clinical settings. Nursing managers should also facilitate interdisciplinary coordination among nurses, physicians, biomedical engineers, and other relevant professionals for alarm review, equipment oversight, and resolution of recurring alarm‐related problems.

At the organizational level, alarm data, adverse events, equipment performance, and relevant quality indicators should be reviewed regularly to inform education, policy adjustment, and quality improvement. Further research is needed to develop and validate comprehensive measures of alarm management competence and to examine how competence develops across clinical settings and career stages. Such evidence could support the incorporation of alarm management competence into nursing competency frameworks, training standards, and quality evaluation systems.

## 5. Conclusions

This concept analysis systematically identified the core attributes, antecedents, and consequences of alarm management competence in clinical nursing practice. The proposed conceptual framework provides a theoretical foundation for future research and offers practical guidance for nursing education, workflow improvement, and technological innovation in alarm management. Further research is needed to examine how alarm management competence is expressed across different clinical settings and to evaluate the effectiveness of targeted interventions. Strengthening alarm management competence may contribute to improved patient safety and enhanced nursing practice in increasingly complex care environments.

## Author Contributions

Bingyu Li: conceptualization, methodology, formal analysis, investigation, data curation, writing–original draft preparation, and writing–review and editing. Liqing Yue: methodology, validation, formal analysis, investigation, writing–review and editing, and supervision. Weizhen Zou: conceptualization and writing–review and editing.

## Funding

This work was supported by the General Program of National Natural Science Foundation of China (NSFC) (72174209), Hunan Province Undergraduate Teaching Reform Research Project (2024087), and Research Project on Educational and Teaching Reform of Central South University (2024jy174).

## Consent

The authors have nothing to report.

## Conflicts of Interest

The authors declare no conflicts of interest.

## Supporting Information

Additional supporting information can be found online in the Supporting Information section.

## Supporting information


**Supporting Information 1** Supporting file 1: PRISMA‐ScR Checklist.

**Supporting Information 2** Supporting file 2: Search strategies used in different databases.

**Supporting Information 3** Supporting file 3: Evidence Matrix for the Attributes, Antecedents, Consequences, and Empirical Referents of Alarm Management Competence.

**Supporting Information 4** Supporting file 4: Surrogate Terms of Alarm Management Competence.

## Data Availability

The data that support the findings of this study are available from the corresponding author upon reasonable request.
