# Supplementary material for: Alarm Management Competence in Clinical Nursing Practice: An Evolutionary Concept Analysis
Source: J Nurs Manag. 2026 Sep 17;2026:3155022. doi: 10.1155/jonm/3155022 (PMC13586673; doi:10.1155/jonm/3155022)
Supplement: Supplementary file 1 [file JONM-2026-3155022-s001.docx]

**Supplementary file 3. Evidence Matrix for the Attributes, Antecedents, Consequences, and Empirical Referents of Alarm Management Competence**

| **No.** | **Study** | **Study design** | **Evidence relevant to alarm management competence** | **Attributes** | **Antecedents** | **Potential consequences / conceptual associations** | **Empirical referents or operational indicators** | **Evidence-overlap note** |
| --- | --- | --- | --- | --- | --- | --- | --- | --- |
| 1 | Winters et al., 2017 | Review | Synthesized approaches to clinical alarm management, including alarm reduction, prioritization, response processes, and strategies addressing alarm fatigue | K, P, D, T, R | A1, A4, A5 | C1, C2, C3 | Alarm burden, response processes, alarm-management strategies | Review-level evidence; primary-study overlap considered during synthesis |
| 2 | Lu et al., 2024 | Qualitative study | Described ICU nurses’ alarm-management coping strategies, including alarm recognition, prioritization, individualized management, clinical judgement, and response | K, P, D, T, C, R, PD | A2, A3, A4 | C1, C2, C3 | Qualitative descriptions of alarm prioritization, individualized settings, and response strategies | Primary study; also included in later qualitative review(s) |
| 3 | Yue et al., 2017 | Systematic review | Synthesized effects of nurse education and training on clinical alarm response and management | K, P, D, T | A2, A6 | C1, C2 | Knowledge, response, practice, and training-related outcomes | Review-level evidence; overlapping primary studies were not counted as independent evidence |
| 4 | Wang et al., 2023 | Cross-sectional descriptive study | Examined ICU nurses’ perceptions and practices regarding clinical alarms, including knowledge, alarm recognition, and management practices | K, P, D, T | A2, A3 | C1 | Questionnaire-based perceptions and alarm-management practices | Primary study |
| 5 | Graham & Cvach, 2010 | Quality improvement | Implemented standardized physiologic-monitor use and nuisance-alarm reduction strategies | T, P, D | A4, A5 | C1, C2, C3 | Alarm frequency, nuisance alarms, monitor settings, process indicators | Primary QI study; cited in subsequent reviews |
| 6 | Bosma & Christopher, 2023 | Quality improvement | Implemented a unit-based alarm-management bundle for critical care nurses | K, T, C, R, PD | A1, A2, A4, A5 | C1, C3 | Bundle adherence, alarm-related process indicators, staff outcomes | Primary study; included in Yang et al. review |
| 7 | Ruppel et al., 2019 | Qualitative study | Explored nurses’ clinical reasoning during physiologic-monitor alarm customization and integration of alarm information with patient context | K, P, D, T, C | A3, A4, A6 | C2 | Qualitative evidence of alarm-customization reasoning and judgement | Primary study; included in He et al. review |
| 8 | Allan et al., 2017 | Quality improvement | Used alarm data to implement alarm-reduction interventions in a cardiovascular surgical ICU | P, T, D | A4, A5 | C2, C3 | Alarm counts, alarm characteristics, intervention/process outcomes | Primary QI study; included in Yang et al. review |
| 9 | Konkani et al., 2012 | Review | Examined medical-device alarm management and environmental noise reduction | P, T | A5, A6 | C1, C3 | Alarm/noise indicators and system-level strategies | Review-level evidence |
| 10 | Phillips, 2006 | Review | Discussed complexity of clinical alarms, prioritization, interpretation, and practical alarm-management requirements | K, P, D, T | A4, A5 | C2, C3 | Conceptual and practice-based indicators | Review-level evidence |
| 11 | Phillips et al., 2020 | Tool-development / educational toolkit | Conceptualized physiologic-monitor use and alarm-system safety in terms of knowledge, skills, clinical reasoning, and attitudes | K, P, D, T, C, R, PD | A2, A4 | C1, C2 | Knowledge-, skill-, and reasoning-oriented educational/evaluation framework | Instrument/toolkit-level evidence |
| 12 | Sowan et al., 2017 | Cross-sectional study | Assessed nurses’ competence in physiologic-monitor use, including hardware, connectivity, alarm management, appropriate monitoring, and advanced functions | K, P, T | A2, A3, A5 | C1, C3 | Nurse Competence on Physiologic Monitors Use Survey | Primary measurement study |
| 13 | Chen et al., 2023 | Systematic review | Identified determinants of medical-equipment alarm fatigue among practicing nurses, including competence-related, organizational, technological, and workload factors | K, P, T, R | A1, A3, A4, A5, A6 | C1 | Alarm-fatigue and alarm-management-related measures across included studies | Review-level evidence; overlapping primary evidence not double-counted |
| 14 | Nyarko et al., 2023 | Integrative review | Synthesized educational interventions aimed at improving nurses’ alarm-management-related knowledge and reducing alarm fatigue | K, P, T, R, PD | A2 | C1 | Knowledge, practice, and alarm-fatigue outcomes | Review-level evidence |
| 15 | Xu et al., 2025 | Meta-synthesis and systematic review | Synthesized ICU nurses’ alarm-response experiences and strategies for managing alarm fatigue | P, D, T, C, R | A3, A4, A5, A6 | C1, C2, C3 | Qualitative manifestations of response, coping, and alarm-management strategies | Review-level evidence; overlap with included primary qualitative studies considered |
| 16 | Movahedi et al., 2023 | Grounded theory study | Explored processes underlying alarm fatigue and nurses’ coping and response strategies in ICU settings | P, D, R | A3, A6 | C1, C2 | Grounded-theory categories related to alarm fatigue, coping, and response | Primary qualitative study |
| 17 | Uçak et al., 2025 | Mixed-methods study | Examined alarm-fatigue levels and nurses’ strategies for reducing fatigue in adult ICUs | P, T, R, PD | A3, A6 | C1, C3 | Alarm Fatigue Scale and qualitative strategy data | Primary mixed-methods study |
| 18 | Yang et al., 2025 | Scoping review | Synthesized ICU alarm-management strategies, including individualized alarm settings, education, standardized device use, response protocols, and multidisciplinary management | K, P, D, T, C | A2, A4, A5 | C1, C2, C3 | Nurses’ alarm-management ability, alarm frequency, alarm-response and system indicators | Review overlaps with Bosma et al. and Allan et al.; duplicated primary evidence not treated as independent support |
| 19 | Chen et al., 2025 | Evidence-based pre–post implementation study | Addressed alarm generation, recognition, response, threshold setting, equipment operation, and alarm-management practice in anesthesia/perioperative staff | K, P, D, T, C | A2, A4, A5 | C1, C2 | Knowledge and evidence-based practice measures, Alarm Fatigue Scale, adverse alarm-management outcomes | Independent implementation study; review sources used within intervention development were not separately weighted |
| 20 | Kim & Yoo, 2025 | Quasi-experimental controlled study | Evaluated VR-based education targeting ventilator-alarm recognition, interpretation, patient assessment, clinical reasoning, and psychomotor performance | K, P, D, T | A2 | C1, C2 | Knowledge, self-efficacy, clinical reasoning, learning immersion | Independent primary study |
| 21 | Kibar & Özsaban, 2025 | Quasi-experimental pre–post study | Examined alarm-management knowledge, behaviors, alarm customization, response, and alarm fatigue after training | K, P, D, T, R | A2 | C1 | Monitor Alarm Management Knowledge Test, behavioral observation, Alarm Fatigue Scale | Independent primary study |
| 22 | Alkubati et al., 2025 | Cross-sectional path analysis | Examined emergency nurses’ alarm knowledge, perceptions, alarm-management practices, and perceived obstacles | K, P, T | A1, A3, A5, A6 | C1, C2 | Knowledge, perception, practice, and obstacle questionnaires | Independent primary study |
| 23 | Li et al., 2025 | Quasi-experimental repeated-measures study | Evaluated a popular-opinion-leader educational intervention targeting alarm-management knowledge, behavioral intentions, prioritization, and alarm fatigue | K, P, D, R | A2, A4 | C1 | Alarm Management Knowledge Test, Alarm Management Behavioral Intention Questionnaire, Alarm Fatigue Scale | Independent primary study |
| 24 | He et al., 2026 | Systematic review of qualitative evidence | Synthesized alarm perception, situated clinical judgement, response strategies, contextual influences, training needs, and autonomy | P, D, C, R | A2, A3, A4, A5, A6 | C1, C2, C3 | Qualitative manifestations of perception, judgement, response, and coping | Includes Lu et al. and Ruppel et al.; overlapping primary evidence not counted independently |
| 25 | Huang et al., 2026 | Qualitative descriptive study | Characterized ventilator-alarm management as recognition, risk prioritization, interpretation, bedside troubleshooting, role-boundary negotiation, and interprofessional escalation | K, P, D, T, C | A2, A3, A4, A5, A6 | C2, C3 | Interview-based manifestations; proposed future observation, alarm-log, response-time, and simulation measures | Independent primary qualitative study |
| 26 | You & Moon, 2026 | Single-group pre–post simulation study | Evaluated repeated-exposure simulation targeting ventilator-alarm recognition, patient assessment, clinical judgement, alarm-limit setting, intervention, and communication | K, P, D, T, C | A2, A3 | C1, C2 | Creighton Competency Evaluation Instrument, ventilator-alarm knowledge test, alarm-management subscore, patient-safety subscore, learning self-efficacy | Independent primary study |
| 27 | Jepsen et al., 2018 | Practice alert | Provided evidence-based recommendations for alarm management in acute care, including individualized alarm settings, appropriate ECG electrode and pulse-oximetry sensor application, alarm-system education, and interprofessional review of alarm practices | K, D, T, C | A2, A4, A5 | C1, C2, C3 | Individualized alarm settings, ECG electrode application, pulse-oximetry sensor placement, alarm review, education and competency-related practices | Authoritative professional guidance; formally published AACN Practice Alert |
| 28 | Sandau et al., 2017 | Scientific statement / practice standards | Provided updated practice standards for hospital ECG monitoring, including appropriate monitoring, alarm settings and management, staff education, documentation, and interdisciplinary implementation | K, D, T, C | A2, A4, A5 | C2, C3 | ECG-monitoring indications, alarm settings, staff education, documentation, and monitoring practices | Authoritative professional statement; formally published AHA Scientific Statement |

Note: K, Foundational Clinical Knowledge for Alarm Management; P, Alarm Perception and Discrimination; D, Clinical Decision-Making; T, Technical Skills for Alarm Equipment Operation; C, Interprofessional Collaboration and Communication; R, Alarm Fatigue Resilience; PD, Professional Development Capacity. A1, Individual Traits and Motivation; A2, Education and Training; A3, Clinical Practice and Experience Accumulation; A4, Organizational and Team Environment; A5, Equipment and Technological Infrastructure; A6, Human Resources and Work Environment. C1, Nurse-related Outcomes; C2, Patient-related Outcomes; C3, Organizational/System-related Outcomes. Where reviews and their underlying primary studies were both included, overlapping evidence was identified and was not treated as additional independent support. PD was coded only when the evidence reflected continued learning, knowledge or skill renewal, or adaptation to evolving alarm-management practices, rather than participation in education or training alone.
