# Supplementary material for: Alarm Management Competence in Clinical Nursing Practice: An Evolutionary Concept Analysis
Source: J Nurs Manag. 2026 Sep 17;2026:3155022. doi: 10.1155/jonm/3155022 (PMC13586673; doi:10.1155/jonm/3155022)
Supplement: Supplementary file 2 [file JONM-2026-3155022-s002.docx]

**Supplementary file 4**

**Surrogate Terms of Alarm Management Competence**

| **Author(s) year** | **Surrogate Terms** | **Relative description** |
| --- | --- | --- |
| Wang et al., 2023 | ability of alarm management;  alarm management ability | - “It is imperative to establish a safe alarm management system, improve ICU nurses' awareness and **ability of alarm management** to improve patients' safety.” - “With the rapid development and updating of medical equipment and instruments, managers should constantly improve the training mechanism, strengthen the training and assessment of nurses on alarm systems, and further improve the **alarm management ability** of ICU nurses.” |
| Uçak et al., 2025 | alarm management competence;  alarm management competency;  alarm management skills | - “Among the alarm fatigue prevention strategies that emerged in our study, “mentor nurses' positive alarm management approaches, developing **alarm management competence** and taking responsibility, and establishing routine working hours in a way that care is not interrupted” constitute new approaches in addition to existing alarm management interventions.” - “In this study, participants emphasised the importance of developing **alarm management competency** and taking responsibility for reducing alarm fatigue.” - “One participant stated that mentor nurses acted as role models for their novice colleagues in acquiring **alarm management skills** and that they first did the right interventions themselves to show the novices.” |
| Ruppel et al., 2019 | alarm management skills | - “New nurses usually need explicit instruction on customisation practices and general **alarm management skills**, and to have those practices reinforced by the unit culture.” |
| Movahedi et al., 2023 | alarm management behavior | - “The **alarm management behaviours** of nurses should be also considered for the assessment of nonactionable alarms and alarm fatigue.” |
| Phillips, 2006 | competency on clinical alarms | - “Box 3. Development of **competency on clinical alarms**:   Identifies clinically significant alarms within the practice environment; Explains need for alarms to patient and family; Stratifies responsibility for: Setting clinical alarms Responding to clinical alarms; Collaborates with other staff to establish a tiered response to alarms; Optimizes patient placement for audibility, especially of standalone alarms; Participates with clinical engineering in the testing of alarms; Responds appropriately to any clinical alarm within the practice environment.” |
| Phillips et al., 2020 | knowledge, skills, and attitude of physiologic monitor use and alarm management | - “DESCRIPTION OF THE TOOLKIT: This educational toolkit is intended for nurse leaders who wish to improve alarm systems safety in acute and critical care and telemetry units where physiologic monitors are in continuous use. Nurse leaders must ensure that nurses operating physiologic monitors and analyzing the resulting clinical data have the necessary **knowledge, skills, and attitude (KSA) to safely operate the monitors and successfully manage alarms.** The components of the educational program include (1) **KSA of physiologic monitor use and alarm management** (Table 1), (2) scenario-based learning model to support the KSA necessary for safe monitor use and alarm management (Table 2), and (3) a framework for evaluating the educational program (Table 3).” - “Using “Physiologic Monitor Use and **Alarm Management Guide: Knowledge, Skills, and Attitude** for Registered Nurses” (Table 1), 80% of the nurses who attended the educational session report they are confident they can “apply the skill they have learned” (column 3 in Table 1) (domain: confidence).” |
| Graham & Cvach, 2010 | alarm management competency | - “As a result of the positive effects of retraining, a skills checklist for **alarm management competency** (Appendix 2, www.ajcconline.org) is now used on the unit to educate all new nurses about the monitor system and to ensure that they are knowledgeable about monitors and alarm management.” |
| Lu et al., 2024 | ability to manage alarms;  professional alarm management ability;  alarm management skills;  alarm management ability;  alarm management capabilities. | - “Thus, enhancing nurses’ awareness and **ability to manage alarms** is crucial for reducing alarm fatigue and risks, thereby improving patient safety.” - “Nurses expressed the importance of accurately identifying medical device alarms and emphasized using clinical cases to improve **professional** **alarm management ability** by enhancing sensory perception and making careful distinctions, learning from incidents and remaining vigilant, and modulating setting the alarm threshold by professional judgment to minimize the false alarm.” - “This study provides a foundation for developing targeted training programs that focus on enhancing nurses’ **alarm management skills**, thereby improving patient outcomes and reducing the risk of alarm fatigue.” - “However, unlike previous studies, our research highlights that this skill development is not solely dependent on technical training but is also significantly influenced by continuous experiential learning and reflection, a concept supported by Cvach, who discussed the importance of ongoing education and hands-on working experience in improving **alarm management ability**.” - “This observation aligns with the conclusions of Milhomme and Pomerleau, who identified reflective clinical practice as a key factor in improving **alarm management** **capabilities.”** |
| Xu et al., 2025 | skills in alarm management;  alarm management behaviors | - “They advocate for utilizing practical opportunities in their work environment and peer education to acquire **skills in alarm management**.” - “Combining intelligent alarm management tools or systems with evidence-based interventions effectively improves nurses’ **alarm management behaviors**.” |
| Chen et al., 2023 | alarm management ability; | - “It is suggested that nursing leaders adopt the alarm management guidelines for clinical training, so as to improve the **alarm management ability** of nurses and reduce the alarm fatigue of nurses.” |
| Bosma & Christopher, 2023 | alarm management skills;  alarm management competency | - “The resulting inadequate **alarm management skills** and low confidence among nurses with regard to safely navigating these monitoring systems have led to increases in the number of alarms and corresponding worsening alarm fatigue among critical care nurses.” - **“Alarm management competency** was given needed attention, and problems were addressed to enable practice improvement.” |
| Sowan et al., 2017 | alarm management skills;  nurse competence on alarm management | - “To guide our initiatives and achieve Phase II of TJC’s clinical alarm systems safety NPSG, we created and validated a structured list of required **alarm management skills** and surveyed ICU nurses on their competence level on using physiologic monitors.” - “The survey used in this study assessed level of perceived competence in using Philips physiologic monitors and consisted of 5 subscales with a total of 59 items. The subscales are admit, discharge, and transfer patient (5 items), hardware and connectivity (6 items), **alarm management** (19 items), appropriate monitoring (24 items), and advanced/specialized functions (5 items).” |

**REFERENCES**

Bosma, S., & Christopher, R. (2023). Implementing a Unit-Based Alarm Management Bundle for Critical Care Nurses. *Critical Care Nurse*, *43*(2), 36–45. https://doi.org/10.4037/ccn2023418

Chen, S., Zhi, H., Wu, S., Jiang, R., Zhang, H., Zhang, H., & Zhang, H. (2023). Determinants of Medical Equipment Alarm Fatigue in Practicing Nurses: A Systematic Review. *SAGE Open Nursing*, *9*. https://doi.org/10.1177/23779608231207227

Graham, K. C., & Cvach, M. (2010). Monitor Alarm Fatigue: Standardizing Use of Physiological Monitors and Decreasing Nuisance Alarms. *American Journal of Critical Care*, *19*(1), 28–34. https://doi.org/10.4037/ajcc2010651

Lu, S.-F., Kuo, Y.-W., Hung, S.-H., Wu, C.-H., Wang, C.-Y., Chou, S.-S., & Huang, S.-H. (2024). Coping strategies of intensive care units nurses in alarm management: A qualitative research study. *BMC Nursing*, *23*(1). https://doi.org/10.1186/s12912-024-02374-1

Movahedi, A., Sadooghiasl, A., Ahmadi, F., & Vaismoradi, M. (2023). A grounded theory study of alarm fatigue among nurses in intensive care units. *Australian Critical Care*, *36*(6), 980–988. https://doi.org/10.1016/j.aucc.2022.12.004

Phillips, J. (2006). Clinical Alarms: Complexity and Common Sense. *Critical Care Nursing Clinics of North America*, *18*(2), 145–156. https://doi.org/10.1016/j.ccell.2006.01.002

Phillips, J., Sowan, A., Ruppel, H., & Magness, R. (2020). Educational Program for Physiologic Monitor Use and Alarm Systems Safety: A Toolkit. *Clinical Nurse Specialist*, *34*(2), 50–62. https://doi.org/10.1097/nur.0000000000000507

Ruppel, H., Funk, M., Whittemore, R., Wung, S., Bonafide, C. P., & Powell Kennedy, H. (2019). Critical care nurses’ clinical reasoning about physiologic monitor alarm customisation: An interpretive descriptive study. *Journal of Clinical Nursing*, *28*(15–16), 3033–3041. https://doi.org/10.1111/jocn.14866

Sowan, A. K., Vera, A. G., Fonseca, E. I., Reed, C. C., Tarriela, A. F., & Berndt, A. E. (2017). Nurse Competence on Physiologic Monitors Use: Toward Eliminating Alarm Fatigue in Intensive Care Units. *The Open Medical Informatics Journal*, *11*(1), 1–11. https://doi.org/10.2174/1874431101711010001

Uçak, A., Cebeci, F., & Tat Çatal, A. (2025). Nurses’ Alarm Fatigue Levels in Adult Intensive Care Units and Their Strategies to Reduce Fatigue: A Convergent Parallel Design. *Journal of Clinical Nursing*, *34*(5), 1691–1703. https://doi.org/10.1111/jocn.17644

Wang, L., He, W., Chen, Y., Wu, Q., Du, X., Li, Q., & Song, C. (2023). Intensive care unit nurses’ perceptions and practices regarding clinical alarms: A descriptive study. *Nursing Open*, *10*(8), 5531–5540. https://doi.org/10.1002/nop2.1792

Xu, D., Liu, F., Ding, X., Ma, J., Suo, Y., Peng, Y.-Y., Li, J., & Fu, X. (2025). Exploring ICU nurses’ response to alarm management and strategies for alleviating alarm fatigue: A meta-synthesis and systematic review. *BMC Nursing*, *24*(1). https://doi.org/10.1186/s12912-025-03084-y
