# Supplementary material for: Alarm Management Competence in Clinical Nursing Practice: An Evolutionary Concept Analysis
Source: J Nurs Manag. 2026 Sep 17;2026:3155022. doi: 10.1155/jonm/3155022 (PMC13586673; doi:10.1155/jonm/3155022)
Supplement: Supplementary file 4 [file JONM-2026-3155022-s004.docx]

**Supplementary file 2**

**Search strategies used in different databases**

| **Databases** | **Search** | **Query** | **Records Retrieved** | **Total Records** |
| --- | --- | --- | --- | --- |
| **PubMed** | #1 | "clinical alarms"[MeSH Terms] OR "alert fatigue, health personnel"[MeSH Terms] OR "alarm"[Title/Abstract] | 16,101 | **374** |
|  | #2 | "manage*"[Title/Abstract] OR "reduce"[Title/Abstract] OR "response"[Title/Abstract] OR "settings"[Title/Abstract] OR "customization"[Title/Abstract] | 6,222,572 |  |
|  | #3 | "clinical competence"[MeSH Terms] OR "professional competence"[MeSH Terms] OR "competenc*"[Title/Abstract] OR "ability"[Title/Abstract] | 1,510,070 |  |
|  | #4 | #1 AND #2 AND #3 | |  |
| **Web of Science** | #1 | AB= (clinical alarms OR alert fatigue) | 6,960 | **208** |
|  | #2 | AB= (clinical competence OR professional competence OR competenc* OR ability) | 1,996,749 |  |
|  | #3 | AB= (manage* OR response OR reduce OR settings OR customization) | 12,222,688 |  |
|  | #4 | #1 AND #2 AND #3 |  |  |
| **Embase** | #1 | 'clinical alarms':ti,ab,kw OR 'alert fatigue':ti,ab,kw OR alarm:ti,ab,kw | 22,702 | **550** |
|  | #2 | manage*:ti,ab,kw OR response:ti,ab,kw OR reduce:ti,ab,kw OR settings:ti,ab,kw OR customization:ti,ab,kw | 8,555,346 |  |
|  | #3 | competence:ti,ab,kw OR ability:ti,ab,kw OR competency:ti,ab,kw | 1,737,872 |  |
|  | #4 | #1 AND #2 AND #3 | |  |
| **CINAHL** | #1 | XB clinical alarms OR alert fatigue, health personnel OR alarm | 5,923 | **193** |
|  | #2 | XB clinical competence OR professional competence OR competenc* OR ability | 327,065 |  |
|  | #3 | XB manage* OR response OR reduce OR settings OR customization | 1,623,481 |  |
|  | #4 | #1 AND #2 AND #3 | |  |
| **Scopus** | #1 | TITLE-ABS-KEY ( clinical AND alarms OR alert AND fatigue OR alarm ) | 10,901 | **402** |
|  | #2 | TITLE-ABS-KEY ( competenc* OR ability ) | 3,739,258 |  |
|  | #3 | TITLE-ABS-KEY ( manage* OR response OR reduce OR settings OR customization ) | 19,669,750 |  |
|  | #4 | #1 AND #2 AND #3 | |  |
| **TOTAL** | | | | **1,727** |
